# Sleep and spa therapies: What is the role of balneotherapy associated with exercise? A systematic review

**DOI:** 10.3389/fphys.2022.964232

**Published:** 2022-08-10

**Authors:** Lucia Castelli, Letizia Galasso, Antonino Mulè, Andrea Ciorciari, Francesca Fornasini, Angela Montaruli, Eliana Roveda, Fabio Esposito

**Affiliations:** ^1^ Department of Biomedical Sciences for Health, University of Milan, Milan, Italy; ^2^ GB Hotels, Abano Terme, Italy; ^3^ IRCCS Istituto Ortopedico Galeazzi, Milan, Italy

**Keywords:** quality of life, thermal effect, mud therapy, pain relief, thermoregulation, relaxation, osteoarthritis, musculoskeletal pain

## Abstract

Balneotherapy and exercise are potential factors influencing sleep through several physiological pathways and relaxing effects. This review aims to assess whether balneotherapy can improve sleep quality in concomitance or not with exercise. The research was conducted on *Medline*, *Scopus*, *PubMed, Web of Science*, and *Cochrane Library* databases. The current review followed PRISMA reporting guidelines and involves twenty-one articles grouped into four sections based on the characteristics of the balneotherapy protocol: 1.a *Balneotherapy–thermal water immersion alone* (five studies); 1.b *Balneotherapy–thermal water immersion with other spa treatments* (six studies); 2.a *Balneotherapy and physical exercise–balneotherapy and out-of-the-pool physical exercise* (eight studies); 2.b *Balneotherapy and physical exercise–balneotherapy and in-pool physical exercise* (three studies). Apart from healthy or sub-healthy subjects, patients recruited in the studies were affected by fibromyalgia, ankylosing spondylitis, osteoarthritis, musculoskeletal pain, subacute supraspinatus tendinopathy, and mental disorders. Duration, number of sessions, and study protocols are very different from each other. Only one study objectively evaluated sleep, whereas the others used subjective sleep assessment methods. Eight studies considered sleep as a primary outcome and ten as secondary. Sixteen out of twenty-one studies described improvements in self-perceived sleep quality. Thus, balneotherapy associated with other spa treatments and physical exercise seems to be effective in improving self-perceived sleep quality. However, the miscellany of treatments makes it difficult to discern the isolated effects of balneotherapy and physical exercise. Future studies should consider using an objective sleep assessment method and describing the pathways and physiological mechanisms that could provoke sleep changes during balneotherapy treatments.

## 1 Introduction

Sleep is a physiological process, defined as a state of rest as opposed to wakefulness, characterized by the total or partial suspension of consciousness. During sleep, there is a slowing of the autonomic functions and a partial interruption of the relationships between subject and environment. Sleep, which is essential for restoring the organism, is an active process involving the interaction of different central and autonomic nervous system components (Chokroverty, 2010; Krueger et al., 2016). Sleep is split into two functional- and control-independent states: 1) Non-rapid eye movements-NREM sleep, subsequently subdivided into four stages (NREM stages 1–4); 2) Rapid eye movements-REM sleep. The NREM and REM sleep is characterized by different modifications in physiological variables, generally in the opposite way. NREM sleep is characterized by slow-wave activity, lowered heart rate and breathing, reduced muscle tone, and absent eye movements. In contrast, REM sleep shows low voltage fast electroencephalogram activities mixed with distinctive saw-tooth waves, swings in blood pressure and heart rate, absent muscle tone, and typical rapid eye movements (Chokroverty, 2010).

There are different tools for sleep evaluation, usually defined as objective or subjective methods. Among the objective ones, polysomnography is a clinical method able to detect the duration and the characteristics of sleep stages and phases (Kinoshita et al., 2021), whilst actigraphy can objectively record the 24-h motor activity providing the assessment of the sleep-wake cycle in the long term (Ancoli-Israel et al., 2003). The subjective methods include a large variety of questionnaires, of which the most widely used is the Pittsburgh Sleep Quality Index (PSQI) (Mollayeva et al., 2016), a self-reported questionnaire describing various domains of sleep (Buysse et al., 1989).

The characteristics of nocturnal sleep are fundamental indicators of the quality of life: the quantity and quality of sleep represent essential factors that can positively or negatively influence the individual psycho-physical abilities and health (Ohayon et al., 2017; Castelli et al., 2022). Regular and efficient sleep derives from the interaction between homeostatic and circadian processes, determining the so-called sleep-wake circadian rhythm or cycle. The pacemaker of the sleep-wake cycle is the suprachiasmatic nucleus (SCN), which acts as a circadian synchronizer of several physiological variables involved in sleep induction and regulation. Circadian and hormonal stimuli promote sleep onset and, after the sleep period, other circadian, hormonal, and, in modern society, also external stimuli promote sleep end and awakening (Hastings et al., 2003; Sack et al., 2007a; Sack et al., 2007b; Montaruli et al., 2021).

The external environment influences, i.e., work activity, exercise, stress, and physical and psychological disorders, affect the sleep process, resulting in potential sleep inducers or disturbers (Chokroverty, 2010; Almojali et al., 2017; Dolezal et al., 2017; Boivin et al., 2022). From this point of view, the spa environment and treatments could play a decisive role in night sleep regulation since the spa therapies may be able to modify the physiological variables involved in sleep induction.

Balneotherapy is the immersion in natural water at the temperature of 36°C–38°C with a mineral content of at least 1 g/L. Mud application over the skin is called mud therapy, while spa therapy refers to different interventions in spa resorts and does not necessarily involve thermal water, such as physical exercise or massage (Fioravanti et al., 2017). The balneotherapy treatment involves immersing a subject in waters or mud to produce an improving effect on health. Traditionally, balneotherapy is employed for several medical reasons: musculoskeletal pain, fibromyalgia, rheumatoid arthritis, and dermatological, pulmonary obstructive and peripherical vascular pathologies (Antonelli et al., 2021; Gebretsadik et al., 2021). In recent years, hypertension, dyslipidaemia, diabetes, obesity, and pathologies characterized by impaired endothelial function also seem to benefit from balneotherapy (Qiu et al., 2014; Fioravanti et al., 2015; De Moraes Silva et al., 2019; Yuan et al., 2019). Indeed, the alternating hot and cold baths positively affect the cardiovascular system, mainly acting on microcirculation (Şaş et al., 2016). The thermal mineral waters and mud show their effects through mechanical, chemical and thermal changes, creating adaptative responses in the autonomic nervous, endocrine, immune, and thermoregulation system (Gutenbrunner et al., 2010). The homeostasis between all these systems is relevant to maintaining regular sleep physiology.

One of the possible links between sleep and balneotherapy could be thermoregulation, which is a crucial component of the sleep-wake cycle. The core body temperature, resulting from heat production and loss, is influenced by environmental and behavioural factors (Parsons, 2014). The optimal thermal range is achieved by altering skin blood flow, hormone levels, and sweating to maintain homeostasis. Body thermoregulation is active during all sleep stages, but is significantly reduced during REM sleep (Bach et al., 2002; Amici et al., 2008). Indeed, according to the circadian cycle, the core body temperature during the day is maintained usually around 37°C, while during the evening, it drops gradually in preparation for sleep (Kräuchi and Deboer, 2011). The drop in core body temperature promotes sleepiness and melatonin production, which signals the body to prepare for sleep. Core temperature decrease in readiness for sleep is principally achieved by a rise in skin blood flow and peripheral heat loss, culminating in higher peripheral skin temperature (Rogers et al., 2007).

Changes in thermoregulation in the hours preceding bedtime could affect sleep, making it better or worse. Indeed, recently some authors have suggested that, as for core body temperature, even small changes in skin temperature are able to affect the circadian thermoregulation system and sleep (Matsui et al., 2016). In this context, hot water immersion induces physiological changes, such as skin vasodilation, increased blood flow and decreased core body temperature, which is a compensatory process following the enhanced body temperature due to hot water. All these physiological changes could lead to modifications in the sleep-wake patterns (An et al., 2019). The influence of balneotherapy on sleep could also depend on the time of day it is administered.

Physical exercise is sometimes included in spa treatments in or out of the water (hydrokinesis therapy). In general, outside of the thermal environment, the role of exercise as a means to improve sleep is recognized throughout the lifespan. Regular exercise lasting at least 12 weeks is, on the one hand, described to decrease NREM stage 1 and, on the other hand, increase REM sleep latency, sleep continuity, and sleep efficiency (Mendelson et al., 2016). Vanderlinden and colleagues’ review (2020) demonstrates that physical exercise significantly improves sleep quality, latency, duration, efficiency, and daytime functioning (Vanderlinden et al., 2020). The role of exercise in sleep is also strengthened because it can mimic body cooling in preparation for rest. Indeed, body temperature increases during exercise, and afterwards, it drops through dissipation mechanisms, including peripheral vasodilation. The similarities between the changes in body core temperature during/after exercise and before falling asleep could help signal to the brain that it is time to fall asleep (Driver and Taylor, 2000).

Based on these considerations, this review aims to study the effect of balneotherapy and spa therapies, particularly physical exercise, on sleep characteristics, in order to assess if these treatments may be considered valuable and efficient tools in sleep management.

## 2 Materials and methods

### 2.1 Search strategy

The current review follows the PRISMA reporting guidelines (Page et al., 2021) and the PICO model for the research question: 1) Population/Problem: healthy and unhealthy subjects; 2) Intervention/Exposure: balneotherapy and/or physical exercise protocols; 3) Comparison: control group, regular medications, other spa treatments, immersion in non-thermal water, physical exercise alone; 4) Outcome: sleep quality and quantity.

Different databases (*Medline*, *Scopus*, *PubMed*, *Web of Science*, and *Cochrane Library*) were searched to find relevant and potential articles to be included in the review. The keywords were: balneotherapy, spa treatments, thermal treatments, sleep, quality of life, physical activity, and exercise combined with #AND and/or #OR. No languages or time restrictions were applied; however, we considered only articles published or available in English. Furthermore, we looked for additional studies in each article’s bibliography. We included only published studies and excluded conference abstracts, proceedings, and publications other than articles published in impacted journals or books. The last article research was run on the 21st of June 2022.

### 2.2 Eligibility criteria

The article’s research focused on studies involving subjects in balneotherapy and spa treatments for at least 1 week. Subjects must have been evaluated in sleep parameters as a first or secondary outcome before and after treatments and be all adults or over 18 years old. Studies were included if either sleep parameters changed or not after the protocols. Sleep evaluations were accepted in several modalities: *ad-hoc* questions on sleep quality and/or problems, questions as a part of broader questionnaires, scales for sleep evaluation, questionnaires designed explicitly for sleep assessment, actigraphy, and polysomnography. No restrictions on the type of the study design were applied.

Balneotherapy protocols using only non-thermal water (e.g., tub or hot water without any specification about its thermal properties or hydrotherapy) were excluded from this review. Balneotherapy protocols could have included different treatments, e.g., water immersion, massages, mud packs, spa steam foot, thermal swimming pool, etc.

### 2.3 Extracted data

A total of *n* = 2,166 articles were identified from the databases; *n* = 1,453 articles were excluded because of duplicates, and *n* = 523 for other reasons. Thus, *n* = 190 records were screened, and *n* = 57 were assessed for eligibility. Of these, *n* = 26 were excluded because they did not use thermal water, *n* = 3 because they reviewed articles not assessing sleep in association with balneotherapy, *n* = 3 because they involved treatments other than balneotherapy, *n* = 6 because they did not consider sleep not among the outcomes. Thus, *n* = 19 articles were selected from databases, and *n* = 2 articles were identified from citation searching, making *n* = 21 articles suitable for the current review.

Extracted data were: patient diagnosis, balneotherapy treatments, eventual treatments in addition to balneotherapy, duration of each treatment, number of sessions and the protocol, the method for sleep assessment, sleep baseline and follow-up values, the number of subjects and their eventual randomization.

Studies were grouped and presented in two sections based on the balneotherapy protocols in association or not with physical exercise:

1. Balneotherapy
  a. Thermal water immersion alone;
  b. Thermal water immersion with other spa treatments;
2. Balneotherapy and physical exercise
  a. Balneotherapy and out-of-the-pool physical exercise;
  b. Balneotherapy and in-pool physical exercise;

### 2.4 Risk of bias assessment

Two authors independently conducted the article research, assessed the risk of bias and discussed possible doubts or divergences. Disagreements were debated until a consensus was reached.

Depending on the study designs, the bias assessment was carried out using two Cochrane risk-of-bias tools: the RoB-2 toll for randomized clinical trials and the ROBINS-I toll for non-randomized study interventions (Sterne et al., 2016; Sterne et al., 2019). Figures summarising risk-of-bias outcomes have been figured out with the *robvis* tool (McGuinness and Higgins, 2021).

## 3 Results

**FIGURE 1:**
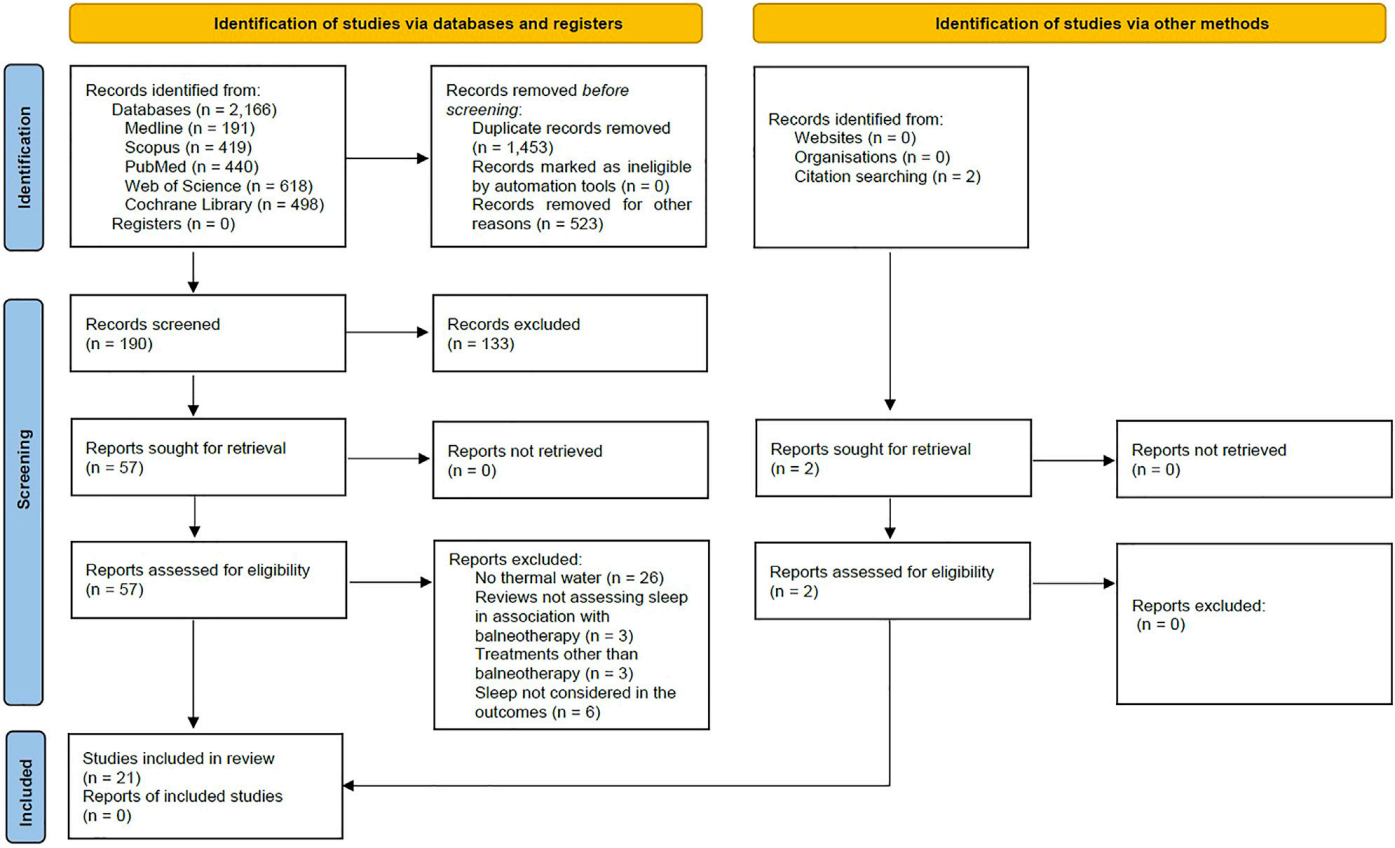
PRISMA flow diagram of the review (Page et al., 2021). For more information, visit: http://www.prisma-statement.org/.

Figure 1 with the PRISMA flow-chart illustrates the studies’ research and selection. The present review involves twenty-one articles that, following the characteristics of the balneotherapy protocol, were grouped into four sections: five studies were included in the *Balneotherapy–thermal water immersion alone* section (Buskila et al., 2001; Neumann et al., 2001; Yang et al., 2018; Koçak et al., 2020; Rapolienė et al., 2020); six studies in *Balneotherapy–thermal water immersion with other spa treatments* section (Dönmez et al., 2005; Sekine et al., 2006; Evcik et al., 2007; Blasche et al., 2010; Koike et al., 2013; Latorre-Román et al., 2015); eight articles in *Balneotherapy and physical exercise–balneotherapy and out-of-the-pool physical exercise* section (Altan et al., 2006; Yurtkuran et al., 2006; Kamioka et al., 2009; Naumann et al., 2020; Stier-Jarmer et al., 2020; Özkuk and Ateş, 2020; Koç et al., 2021; Bestaş et al., 2022), and three studies in the *Balneotherapy and physical exercise–balneotherapy and in-pool physical exercise* section (Altan et al., 2004; Maindet et al., 2021; Bestaş et al., 2022). The study by Bestaş and colleagues (2022) has been included in two different sections since its randomization was based on three arms: 1) Balneotherapy; 2) water-based physical exercise; 3) land-based physical exercise.

The study samples were relatively small and between 13 and 90 participants. Only five studies showed a sample with more than one hundred participants (Sekine et al., 2006; Yang et al., 2018; Rapolienė et al., 2020; Stier-Jarmer et al., 2020; Maindet et al., 2021). The studies included in the review were on healthy or sub-healthy subjects (Sekine et al., 2006; Kamioka et al., 2009; Blasche et al., 2010; Latorre-Román et al., 2015; Stier-Jarmer et al., 2020) and patients affected by different pathologies (mainly chronic pathologies or syndromes, i.e., fibromyalgia (Buskila et al., 2001; Neumann et al., 2001; Altan et al., 2004; Dönmez et al., 2005; Maindet et al., 2021), ankylosing spondylitis (Altan et al., 2006; Bestaş et al., 2022), osteoarthritis (Yurtkuran et al., 2006; Evcik et al., 2007), musculoskeletal pain (Rapolienė et al., 2020; Özkuk and Ateş, 2020), morbid obesity (Koçak et al., 2020), mental disorders (Koike et al., 2013; Naumann et al., 2020) and subacute supraspinatus tendinopathy (Koç et al., 2021)).

In fourteen out of twenty-one studies, the mean age was around 50 years; three studies involved older adults around the 70s or older (Koike et al., 2013; Latorre-Román et al., 2015; Özkuk and Ateş, 2020); three studies reported age classes 18–65 years (Sekine et al., 2006; Yang et al., 2018; Rapolienė et al., 2020), and one study did not specify the mean age of the sample (Altan et al., 2006).

Except for the study by Sekine and colleagues (2006), all the studies set up a balneotherapy intervention protocol, with more than half of the studies (sixteen) showing both an intervention and control (or with other therapies excluding balneotherapy) group (Buskila et al., 2001; Neumann et al., 2001; Altan et al., 2004, 2006; Dönmez et al., 2005; Yurtkuran et al., 2006; Kamioka et al., 2009; Yang et al., 2018; Naumann et al., 2020; Rapolienė et al., 2020; Stier-Jarmer et al., 2020; Özkuk and Ateş, 2020; Koç et al., 2021; Maindet et al., 2021; Bestaş et al., 2022), whereas the remaining studies did not randomize the study sample (Evcik et al., 2007; Blasche et al., 2010; Koike et al., 2013; Latorre-Román et al., 2015; Koçak et al., 2020).

The balneotherapy protocol duration had a minimum length of 10 days (Buskila et al., 2001; Neumann et al., 2001) and a maximum of 24 weeks (Kamioka et al., 2009). However, Sekine and colleagues (2006) study evaluated the individual spa visit frequency for 3 years, but it did not report the mean visit frequency or other values comparable with the other articles (Sekine et al., 2006). The minimum number of balneotherapy treatment sessions was four (Stier-Jarmer et al., 2020), while the maximum was thirty-six (Altan et al., 2004). Also in this case, it was not possible to establish the exact number of treatment sessions in the studies by Sekine and colleagues (2006) and Yang and colleagues (2006) (Sekine et al., 2006; Yang et al., 2018). Most of the articles reported balneotherapy session length of 20 min (Buskila et al., 2001; Neumann et al., 2001; Dönmez et al., 2005; Yurtkuran et al., 2006; Evcik et al., 2007; Kamioka et al., 2009; Koike et al., 2013; Latorre-Román et al., 2015; Koçak et al., 2020; Naumann et al., 2020; Rapolienė et al., 2020; Stier-Jarmer et al., 2020; Özkuk and Ateş, 2020; Koç et al., 2021; Bestaş et al., 2022), but in some cases, it was longer (30 min or more) (Altan et al., 2004, 2006; Blasche et al., 2010; Yang et al., 2018; Maindet et al., 2021).

Sleep was assessed mainly with subjective assessment methods, through *ad hoc* questions about sleep problems (Buskila et al., 2001), number of hours slept (Kamioka et al., 2009) and sleep quality (Yang et al., 2018) in three articles; through specific-sleep items of broader questionnaires in seven articles (*Nottingham Health Profile*—NHP-Sleep (Wiklund, 1990; Altan et al., 2006; Yurtkuran et al., 2006; Evcik et al., 2007; Koçak et al., 2020; Özkuk and Ateş, 2020); *Recovery Stress Questionnaire* (Kallus, 1995; Blasche et al., 2010); *Hamilton Depression Scale* (Williams, 1988; Altan et al., 2004); through the *Visual Analogue Scale* (VAS) (Wewers and Lowe, 1990) method in four articles (Neumann et al., 2001; Dönmez et al., 2005; Rapolienė et al., 2020; Koç et al., 2021); through specific sleep questionnaires in seven articles (*Pittsburgh Sleep Quality Index*—PSQI (Buysse et al., 1989; Sekine et al., 2006; Koçak et al., 2020; Naumann et al., 2020; Maindet et al., 2021; Bestaş et al., 2022); *Oviedo Sleep Questionnaire*—OSQ (Bobes et al., 1998; Latorre-Román et al., 2015); *Insomnia Severity Index*—ISI (Bastien, 2001; Stier-Jarmer et al., 2020). Only one study used actigraphy (Sadeh, 2011; Koike et al., 2013), one of the most utilized objective methods to record and evaluate sleep.

Sleep changes were considered a primary outcome in eight studies (Altan et al., 2004; Sekine et al., 2006; Blasche et al., 2010; Latorre-Román et al., 2015; Yang et al., 2018; Koçak et al., 2020; Özkuk and Ateş, 2020; Bestaş et al., 2022), a secondary outcome in the majority of the studies (Buskila et al., 2001; Dönmez et al., 2005; Altan et al., 2006; Sekine et al., 2006; Yurtkuran et al., 2006; Kamioka et al., 2009; Naumann et al., 2020; Rapolienė et al., 2020; Stier-Jarmer et al., 2020; Koç et al., 2021; Maindet et al., 2021), while it was not specified in three studies (Neumann et al., 2001; Evcik et al., 2007; Koike et al., 2013). Most of the studies evaluated sleep at the beginning and the end of the balneotherapy and at several follow-up times (Buskila et al., 2001; Neumann et al., 2001; Altan et al., 2004, 2006; Dönmez et al., 2005; Yurtkuran et al., 2006; Evcik et al., 2007; Kamioka et al., 2009; Blasche et al., 2010; Koçak et al., 2020; Naumann et al., 2020; Rapolienė et al., 2020; Stier-Jarmer et al., 2020; Özkuk and Ateş, 2020; Maindet et al., 2021; Bestaş et al., 2022); whereas, four studies checked for sleep changes only at the end of the balneotherapy protocol (Koike et al., 2013; Latorre-Román et al., 2015; Yang et al., 2018; Koç et al., 2021).

The studies came from the Eurasia region, with three studies from Japan (Sekine et al., 2006; Kamioka et al., 2009; Koike et al., 2013), nine from Turkey (Altan et al., 2004; Dönmez et al., 2005; Altan et al., 2006; Yurtkuran et al., 2006; Evcik et al., 2007; Koçak et al., 2020; Özkuk and Ateş, 2020; Koç et al., 2021; Bestaş et al., 2022), and the others from China (Yang et al., 2018), Israel (Buskila et al., 2001; Neumann et al., 2001), Lituania (Rapolienė et al., 2020), Austria (Blasche et al., 2010), Germany (Naumann et al., 2020; Stier-Jarmer et al., 2020), France (Maindet et al., 2021), and Spain (Latorre-Román et al., 2015).

Finally, regarding the risk of bias assessment, results for randomized clinical trials are figured out in Supplementary Figure S1. The final judgement for the fifteen randomized clinical trials ranged between some concerns and high overall risk of bias. All studies recorded some concerns judgement in Domain 2 (Bias due to deviations from intended intervention) since the intrinsic characteristics of the study protocol did not allow a double-blind design, and the participants were aware of their allocation group. Furthermore, VAS as a sleep assessment method has been considered inappropriate for evaluating sleep quality. Indeed, due to the nature of the VAS scale, it could be considered a generic instrument able to evaluate only one aspect of sleep and not the several parameters and the complexity characterizing sleep. The six non-randomized intervention studies gathered a final judgement between moderate and low overall risk of bias (Supplementary Figure S2), with Domain 1 (Bias due to confounding) showing, in general, moderate bias since lots of studies did not control for confounding factors.

## 4 Discussion

### 4.1 Balneotherapy

#### 4.1.1 Thermal water immersion alone

The studies analyzed in this session are summarised in Table 1. Two studies involved women with fibromyalgia syndrome (Buskila et al., 2001; Neumann et al., 2001), while the other three recruited people with morbid obesity (Koçak et al., 2020), musculoskeletal pain (Rapolienė et al., 2020), and sub-healthy problems (Yang et al., 2018).

**TABLE 1:** Balneotherapy–Thermal water immersion alone.

Study	N; population type	Age	Study design	Intervention	Comparison(s)	Sleep assessment; relevance of sleep assessment	Follow-up(s)	Sleep results	Significant changes
Neumann et al., 2001	48; women with fibromyalgia	54.6 (IG) 54.3 (CG)	RCT	BT (*n* = 24) 10 sessions; 1 treatment per day; 20 min	Regular medications (*n* = 24)	VAS; n.a	Study end; 1st month; 3rd month	No clear trend	No
Buskila et al., 2001	48; women with fibromyalgia	54.6 (IG) 54.3 (CG)	RCT	BT (*n* = 24) 10 sessions; 1 treatment per day; 20 min	Regular medications (*n* = 24)	Sleep questions; secondary outcome	Study end; 1st month; 3rd month	Reduction trend	No
Koçak et al. (2020)	54; women with morbid obesity	58.4	NRIS	BT 15 sessions; 1 treatment per day; 20 min		PSQI, NHP-Sleep; primary outcome	Study end; 3rd week	A clear trend of improvement in sleep disorders	Yes
Rapoliené et al., 2020	145; subjects with musculo-skeletal pain	18–65	RCT	BT (*n* = 94) 10 sessions; 1 treatment per day; 20 min	Tap water (*n* = 26) No treatment (*n* = 25)	VAS; secondary outcome	Study end; 1st month; 2nd month; 3rd month	Sleep quality improved in all the mineral concentrations groups, with a long-lasting effect in 40 g/L group	Yes
Yang et al., 2018	362; sub-healthy subjects	18–65	RCT	BT (*n* = 223) 5 weeks; 1–3 treatments per week; 30 min	No treatment (*n* = 139)	Sleep questions; primary outcome	Study end	Sleep quality, difficulty in falling asleep, easy awakening, dreaminess, nightmare suffering, and restless sleep improved	Yes

IG, intervention group; CG, control group; RCT, randomised clinical trial; NRIS, nonrandomised intervention study; BT, balneotherapy; VAS, visual analogue scale; PSQI, pittsburgh sleep quality index; NHP-Sleep, Nottingham Health Profile–Sleep; n.a., not available.

The two studies on fibromyalgia syndrome had a similar balneotherapy protocol: all the women stayed at the Dead Sea, with the intervention group (*n* = 24) undergoing baths in sulphur water for 20 min for 10 days, whereas the control group (*n* = 24) merely continued the regular medication. In the study by Neumann and colleagues (2001), sleep, investigated with VAS, did not show any modifications neither at the end of the treatments nor at the two follow-ups after one and 3 months (Neumann et al., 2001). Conversely, in the study by Buskila and colleagues (2001), both groups improved their sleep quality. In the latter study, the authors advanced the hypothesis that the study allocation with a relaxing and less stressful atmosphere could have favoured the improvements in sleep quality (Buskila et al., 2001). The differences in sleep outcomes could be traceable in the different sleep assessment tools.

As mentioned earlier, one of the limitations of the two studies is that the inclusion criteria were restricted to the female sex, which is the same situation detected in the study by Koçak and colleagues (2020) on 54 women with morbid obesity. All the women underwent fifteen thermal water immersions lasting 20 min and filled in the PSQI and the NHP-Sleep. Both scores improved at the end of balneotherapy. The authors concluded that fifteen balneotherapy sessions effectively improved sleep quality, but they did not advance any hypothesis aiming at explaining these changes. Furthermore, the inflammation (C-reactive protein–CRP) and cortisol levels, two factors showing a potential impact on sleep, did not change after balneotherapy (Koçak et al., 2020).

Rapoliené and colleagues (2020) recruited a sample of 154 subjects with musculoskeletal pain and divided them into five groups: the first three (intervention groups) had bathed in hot thermal water with different mineral concentrations (20 g/L, 40 g/L, 60 g/L; *n* = 35, *n* = 28, *n* = 31, respectively), one immersed in tap water (*n* = 26) and the last did not follow any treatments (*n* = 25). All the immersions lasted 20 min and were repeated for 10 days. Sleep was assessed with VAS and significantly improved in all the three mineral-bath groups. Sleep improvements were visible and significant at the end of balneotherapy and after 2 months of follow-up, with the 40 g/L group showing the most lasting improvement even after 3 months of follow-up. In concomitance with sleep, pain and inflammation (CRP) levels also improved in the intervention groups (Rapolienė et al., 2020). The authors supposed that lower inflammation levels (CRP) associated with decreased pain sensitivity could have had beneficial effects on self-perceived sleep quality (Afari et al., 2011).

A more extended balneotherapy protocol, 1–3 thermal water immersion lasting 30 min for 5 months, was set up by Yang and colleagues (2018). The study sample was divided into an intervention (*n* = 139) and a control group (*n* = 223), whose sleep was assessed through the presence or not of four sleep problems: difficulty in falling asleep, easy awakenings, difficulty in falling asleep again after awakening, and dreaminess and nightmare suffering. All four parameters improved from the baseline to the follow-up in the intervention group; additionally, at the end of the balneotherapy protocol, the intervention group showed better sleep parameters than the control group. Even though they did not check body temperature, the authors explained the enhanced sleep quality with the proper adjustments in body temperature after balneotherapy that could have promoted deep sleep. However, the number of balneotherapy sessions was not fixed since the authors suggested having at least one balneotherapy per week, and the total balneotherapy sessions could be different depending on the individuals. Furthermore, the sleep quality in the intervention group at baseline was significantly lower than that of the control group, which could have constituted the basis for a more remarkable and significant improvement in the intervention group’s sleep (Yang et al., 2018).

In conclusion, excluding the studies by Neumann and colleagues (2001) and by Rapoliené and colleagues (2020) because of the use of the VAS scale, which is not wholly appropriate to investigate sleep, the other studies highlighted an improvement in sleep quality after the balneotherapy protocol. Even without physiological, significant correlational changes, and bidirectional effects’ explanation and evidence, balneotherapy consisting of thermal water immersion seems to be effective in improving the self-perceived sleep quality. Causes could be traceable in reduced pain, inflammation, stress, better body temperature regulation, and the relaxing atmosphere of the thermal centres.

#### 4.1.2 Thermal water immersion with other spa treatments

In the current section (Table 2), one study involved women with fibromyalgia syndrome (Dönmez et al., 2005), one recruited patients with knee osteoarthritis (Evcik et al., 2007), two focused on patients with psychological disorders (Blasche et al., 2010) and cognitive impairments (Koike et al., 2013), one enrolled healthy older adults (Latorre-Román et al., 2015) and the last engaged civil servants of the Japanese administration.

**TABLE 2:** Balneotherapy–Thermal water immersion with other spa treatments.

Study	N; population type	Age	Study design	Intervention	Comparison(s)	Sleep assessment; relevance of sleep assessment	Follow-up(s)	Sleep results	Significant changes
Dönmez et al., 2005	30; women with fibromyalgia	43.3 (IG) 43.1 (CG)	RCT	BT and PS or M (*n* = 16) 12 sessions; 2 treatments per day; 20 min	Regular medications (*n* = 14)	VAS; secondary outcome	Study end; 1st month; 3rd month; 6th month; 9th month	Improvement in both groups in sleep disturbance	Yes
Evcik et al., 2007	80; patients with knee osteoarthritis	57 ca	NRIS	BT (*n* = 25) MT (*n* = 29) 10 sessions; 1 treatment per day; 20 min	HPA (*n* = 26) 10 sessions; 1 treatment per day; 20 min	NHP-Sleep; n.a	Study end; 3rd month	Sleep quality improvements in BT and MT groups	Yes
Blasche et al., 2010	65; subjects with occupational burnout	50.4	NRIS	SPA therapies (*n* = 65) 18 sessions; 3–4 treatments per day; 20–40 min		Recovery Stress Questionnaire; primary outcome	Study end; 1st month; 3rd month	Improvement in sleep quality	Yes
Koike et al., 2013	13; patients with cognitive impairment	82.67	NRIS	SF 10 sessions; 1 treatment per day; 20 min		Actigraph; n.a	Study end	No improvement in total sleep time, night-time sleep and night-time sleep efficiency	No
Latorre-Román et al., 2015	52; healthy elderly people	69.7 (IG)	NRIS	SPA therapies 12 sessions; 20 min (BT) + 2 h (other therapies)		Oviedo Sleep Questionnaire; primary outcome	Study end	Clear improvement in sleep quality and insomnia symptoms	Yes
Sekine et al., 2006	3,341; government employees	20–65	CSS	SPA therapies		PSQI; primary outcome	n.a	The more frequent SPA resort users are more likely to have a better quality of sleep	Yes

IG, intervention group; CG, control group; RCT, randomised clinical trial; NRIS, nonrandomised intervention study; CSS, cross sectional survey; BT, balneotherapy; PS, pressure shower: M, massage; MT, mud therapy; HPA, hot pack application; SF, steam foot; VAS, visual analogue scale; PSQI, pittsburgh sleep quality index; NHP-Sleep, Nottingham Health Profile–Sleep; n.a, not available.

The first study, involving 30 women affected by fibromyalgia syndrome, contemplated a protocol with a balneotherapy intervention (*n* = 14) of 2 weeks with thermal water immersion (20 min) and a shower with thermal massage (15 min) or a classical massage (15 min) in addition to regular medications. VAS assessed sleep that significantly improved after the balneotherapy until the ninth month of follow-up (except at the third month of follow-up). However, sleep in the control group (only regular medications; *n* = 16) also improved during the sixth and ninth months of follow-up. The authors did not comment on sleep values in the discussion (Dönmez et al., 2005). However, the concomitant improvements in pain, tender point count and other parameters may suggest that the less annoying fibromyalgia symptoms could less negatively interfere with sleep. As mentioned before, the sleep assessment with VAS leads to carefully considering the results obtained with an unsuitable method.

In the study by Evcik and colleagues (2007), participants were divided into three groups based on the followed treatment: thermal water immersion (*n* = 25); mud pack therapy (*n* = 29); hot-pack therapy (*n* = 26). All the groups underwent ten sessions lasting 20 min, but only the first two treatments were considered balneotherapy interventions. Sleep, assessed through NHP-Sleep, improved in the first two groups, and the enhanced sleep quality was significant until the last follow-up after 12 weeks (Evcik et al., 2007). As previously seen in other articles, sleep improved concomitantly with pain relief. Furthermore, mud pack applications linked to massages could positively influence inflammation (stimulation of anti-inflammatory components and shear stress on macrophages) and body temperature, two factors involved in sleep regulation (Bellometti et al., 1997; Bellometti et al., 2000; Driver and Taylor, 2000; Irwin et al., 2016; Saitou et al., 2018).

In addition to physical illnesses, balneotherapy is also prescribed for stress, psychological and cognitive disorders. In this context, the first study that focused on mental rather than physical impairments was by Blasche and colleagues (2010), who tried to relieve the psychological symptoms, including sleep, of occupational burnout in 65 subjects. The balneotherapy protocol included classical massage, underwater jet massage, tub baths in naturally carbonated mineral water, hot mud packs, and water and relaxation exercise (20–40 min), with 3–4 treatments per day for 18 days. Sleep, assessed with some questions from the Recovery Stress Questionnaire, significantly improved after the balneotherapy treatments and this improvement lasted until 3 months of follow-up (Blasche et al., 2010). Improving sleep was one of the main goals of this study since sleep deficiency is one of the burnout symptoms. Balneotherapy could have been one of the factors involved in sleep quality recovery; however, regain from burnout could also be traceable in respite from work and spontaneous improvements (Westman and Eden, 1997; Leone et al., 2008). Thus, balneotherapy’s primary or single effect cannot be stated with certainty.

The second study involving patients with cognitive impairments was by Koike and colleagues (2013), which is the only one that provided an objective sleep assessment through actigraphy. The current study did not offer a proper thermal water immersion of the entire body, but its protocol consisted of a steam foot spa of 20 min for 12 days. At the end of the treatments, actigraphic parameters of the thirteen elderly participants, such as total sleep time, night-time sleep, and sleep efficiency, did not show any significant improvements (Koike et al., 2013). The results of this article raise two doubts. The first is that 12 days with only a steam foot spa are insufficient to influence sleep or its regulating mechanisms such as body temperature, parasympathetic activity, or inflammation. The second one is that an objective sleep assessment could lead to different and opposite results from a subjective sleep assessment. The literature already reported that the improvements in self-perceived sleep quality could not follow the outcomes of objective sleep assessments (Rogers et al., 2017; Kreutz et al., 2019). The fact that only one study based its results on actigraphic sleep assessment makes the last sentence only a mere supposition.

In addition to physical or mental impaired subjects, balneotherapy is also used in healthy subjects to highlight the additional benefits of thermal-water treatments. In this context, Latorre-Romain and colleagues (2015) evaluated sleep changes (Oviedo Sleep Questionnaire) in a cohort of 52 healthy older subjects after 12 days of balneotherapy. The balneotherapy protocol consisted of hot thermal-, bubble-, and hot tub-baths with underwater hydro jets, thermal jet and shower, inhalations, and thermal muds. After the treatments, participants improved all three sleep dimensions (satisfaction with sleep, insomnia, hypersomnia). The authors also evaluated men and women separately, highlighting some differences; the former did not improve sleep satisfaction, while the latter did not decrease hypersomnia. Even though the authors did not assess body temperature, they supposed that manipulating core body temperature after hot baths could improve the circadian body temperature rhythm, promoting better sleep in the elderly (Latorre-Román et al., 2015).

The study by Latorre-Romain and colleagues (2015) was one of the few studies investigating sleep separately in men and women. In this case, Sekine and colleagues (2006) also made some differences in sleep quality between sexes. The current study tried to find a connection or correlation between the frequency of spa use during the last 3 years (never, 1–2 times, 3–4 times, >5 times) and sleep (PSQI). No information about the balneotherapy protocols was available; however, the extensive study sample (*n* = 3,341) showed that subjects with a higher frequency of spa use were more likely to show better sleep quality. Furthermore, women slept significantly worse than men, and their OR in improving sleep by visiting the spa centres more frequently was higher than that of men (Sekine et al., 2006).

### 4.2 Balneotherapy and physical exercise

#### 4.2.1 Balneotherapy and out-of-the-pool physical exercise

The current section (Table 3) encompasses eight studies in which protocols set up balneotherapy sessions in concomitance with treatments unrelated to the thermal context, i.e., physical exercise protocol alone (Altan et al., 2006; Naumann et al., 2020; Bestaş et al., 2022) or together with other strategies (Yurtkuran et al., 2006; Kamioka et al., 2009; Stier-Jarmer et al., 2020; Özkuk and Ateş, 2020; Koç et al., 2021).

**TABLE 3:** Balneotherapy and physical exercise–Balneotherapy and out-of-the-pool physical exercise.

Study	N; population type	Age	Study design	Intervention	Comparison(s)	Sleep assessment	Follow-up(s)	Sleep results	Significant changes
Yurtkuran et al., 2006	52; patients with knee osteoarthritis	52.9 (IG) 55.5 (CG)	RCT	BT + PE (*n* = 27) 14 sessions; 2 treatments per day; 20 + 20 min	TW + PE (*n* = 25) 14 sessions; 2 treatments per day; 20 + 20 min	NHP-Sleep; secondary outcome	Study end; 3rd month	Improvement in the sleep score in both groups, but superior in the IG.	Yes
Altan et al., 2006	54; patients with ankylosing spondylitis	n.a	RCT	BT + PE (*n* = 28) 21 + 180 sessions; 30 + 30 min	BT (*n* = 26) 180 sessions; 1 treatment per day; 30 min	NHP-Sleep	Study end; 6th month	In the IG, sleep improved after the first, but not after the second follow-up. In the CG, no modification in the sleep parameters	Yes
Bestaş et al. (2022)	60; patients with ankylosing spondylitis	42.3 (BT) 42 (PE)	RCT	BT (*n* = 30) 20 sessions; 20 min	PE (*n* = 30) 20 sessions; 60 min	PSQI	Study end; 3rd month	Sleep improved in both groups in the total score and most but not all domains	Yes
Özkuk and Ateş, (2020)	58; patients with chronic shoulder pain	65 ca	RCT	BT + PT + PE (*n* = 28) 15 sessions; 3 treatments per day; 20 min + n.a. + 15 min	PT + PE (*n* = 30) 15 sessions; 3 treatments per day; n.a. + 15 min	NHP-Sleep; primary outcome	Study end; 1st month	Both groups improved sleep at both follow-ups. IG showed significantly better sleep than CG only at the second follow-up	Yes
Koç et al. (2021)	90; patients with subacute supraspinatus tendinopathy	48.8 (IG) 47.3 (CG)	RCT	BT + PT + PE (*n* = 45) 15 sessions; 3 treatments per day; 20 + 40 + 15 min	PT + PE (*n* = 45) 15 sessions; 2 treatments per day; 40 + 15 min	VAS; secondary outcome	Study end	Both groups significantly improved their sleep quality; the IG group showed a greater improvement	Yes
Naumann et al., 2020	45; patients with depression	48.4	RCT	BT + HTB (*n* = 22) 16 sessions; 2 treatments per day; 20 + 20 min	PE (*n* = 23) 16 sessions; 1 treatments per day; 45 min	PSQI; secondary outcome	2nd treatment week; study end	Improvement in sleep quality in the IG and a weak trend of improvement in the CG	Yes
Kamioka et al., 2009	43; healthy men	41.1 (IG) 46.3 (CG)	RCT	HE + BT + PE (*n* = 22) 12 + 24 + 24 sessions; 2–3 treatments per week; 60 + 20 + 30 min	HE (*n* = 21) 1 single session	Sleep questions; secondary outcome	Study end; 1st year	No results in terms of sleep hours per day	No
Stier-Jarmer et al., 2020	103; farmers	55.3	RCT	SMI (10 sessions × 2 h) + PMR (12 sessions × 0.5/1 h) + PE (10 sessions × 1 h) + BT (4 sessions × 20 min) + TC (*n* = 51)	SMI (10 sessions × 2 h) + PMR (12 sessions × 0.5/1 h) + PE (10 sessions × 1 h) + BT (4 sessions × 20 min) (*n* = 52)	Insomnia Severity Index; secondary outcome	1st month; 6th month; 9th month	The percentage of participants with moderate or severe insomnia decreased after the intervention	Yes

IG, intervention group; CG, control group; RCT, randomised clinical trial; BT, balneotherapy; PE, physical exercise; TW, tap water; HE, health education; PT, physical therapy; HTB, hyperthermic bath; SMI, stress management intervention; PMR, progressive muscle relaxation; VAS, visual analogue scale; PSQI, pittsburgh sleep quality index; NHP-Sleep, Nottingham Health Profile–Sleep; n.a, not available.

Five studies focused on muscular osteo-arthrous pathologies (Altan et al., 2006; Yurtkuran et al., 2006; Özkuk and Ateş, 2020; Koç et al., 2021; Bestaş et al., 2022). The first tried to discover sleep improvement differences in patients with knee osteoarthritis following home-based physical exercise sessions in concomitance with thermal water immersion (intervention group; *n* = 27) or hot water immersion (control group; *n* = 25) for ten sessions ×20 min. Sleep, assessed with the NHP-Sleep, improved in both groups, and the percent change in sleep values was superimposable in the two groups at the end of the study protocol and the 12th week follow-up. Probably, the relaxing spa ambience experienced by both groups could have had a beneficial effect on sleep, independently of the water type for the immersion (Yurtkuran et al., 2006).

The second study by Altan and colleagues (2006) investigated the differences between a balneotherapy protocol in association with a physical exercise protocol (30 min of thermal water immersion for 3 weeks; intervention group = 30 subjects) and physical exercise alone (control group = 30 subjects) in a cohort of patients suffering from ankylosing spondylitis. Through NHP-Sleep assessment, sleep immediately improved in the intervention group after the end of balneotherapy treatments, but the improvements were no longer visible at the twenty-4th-week follow-up. Conversely, sleep in the control group improved only at the twenty-4th-week follow-up. The main conclusions and explanations were that balneotherapy effectively enhanced sleep in the short term, whereas exercise was more successful in bettering sleep in the long term (Altan et al., 2006).

Differently, the recent article published by Bestaş and colleagues (2022) described significant and long-lasting (12 weeks of follow-up) effects on sleep of both balneotherapy (20 sessions lasting 20 min each) and out-of-the-pool physical exercise (20 sessions lasting 60 min each) interventions. More specifically, the PSQI final score and some of its domains (habitual sleep efficiency, sleep disturbances, use of sleep medications, daytime dysfunctions, sleep latency in the balneotherapy group and sleep duration in the physical exercise group) significantly improved at the end of the interventions (4 weeks). Furthermore, they maintained the improvements after 12 weeks of follow-up (Bestaş et al., 2022). The last two studies showed some differences in the intervention protocols that could justify the different outcomes: the study by Altan and colleagues (2006) had a protocol lasting 3 weeks against the four by Bestaş and colleagues (2022); the former opted for a longer follow-up time point (24 weeks), while the latter chose a shorter follow-up time point (12 weeks); finally, sleep assessment methods differed, with the PSQI, chose by Bestaş and colleagues (2022), appearing as more accurate than NHP-Sleep in describing sleep and its domains/parameters (Altan et al., 2006; Bestaş et al., 2022).

Finally, two studies focused on alleviating shoulder pain and reduced mobility. Both flanked the physical exercise to other physical therapies, i.e., Transcutaneous Electrical Nerve Stimulation (TENS), hot pack and ultrasound stimulations (Özkuk and Ateş, 2020; Koç et al., 2021); thus, the conclusions of these studies are less defined and transparent since it is impossible to discern the effect of balneotherapy isolated from the effects of the other treatments. Indeed, in the study by Özkuk and Ateş (2020), the 58 participants with chronic shoulder pain were divided into two groups: the PT (physical therapy) group received TENS and physical exercise; the BT (balneotherapy) group received the same treatments with balneotherapy addition of 20 min per day for fifteen sessions. NHP-Sleep results improved in both groups at the end of the treatments and after the 1-month follow-up. However, sleep improvements in the BT group were more significant than those of the PT group only at the 1-month follow-up (Özkuk and Ateş, 2020).

Notwithstanding similarities in the study protocol and duration, the results by Koç and colleagues (2021) are somehow discordant from those by Özkuk and Ateş (2020). Indeed, in the study by Koç and colleagues (2021), both at the end-study and follow-up, sleep improvements were significant in both groups; furthermore, the BT group’s sleep improvements showed a significant greater delta value than that of the PT group (Koç et al., 2021). Regarding the last study, it is essential to keep in mind that sleep assessment was carried out with VAS and differences in sleep outcomes could be traceable in the inadequate sleep assessment method.

Regarding the use of balneotherapy and physical exercise in mental disorders, the recent study by Naumann and colleagues in 2020 shed light on different sleep changes in subjects with depressive syndrome. The protocol lasted 8 weeks with two weekly sessions; the intervention group did balneotherapy with thermal water immersion in a spa centre and hot water baths at home (20 + 20 min), while the control group did moderate physical exercise sessions (45–50 min). Sleep changes (PSQI) were checked during the second and eighth week of intervention: the intervention group improved sleep at both time points, whereas the control group improved sleep only at the last follow-up. Consequently, sleep was significantly better in the intervention group than in the control group after 2 weeks of intervention; conversely, differences in sleep quality were not more significant after 8 weeks of intervention. Thus, as Altan and colleagues (2006) previously saw, physical exercise seemed to have a more lasting or delayed effect than balneotherapy in depressed subjects (Naumann et al., 2020).

Balneotherapy and physical exercise are two potential interventions for preventing chronic pathologies, such as obesity and metabolic syndrome. In this context, Kamioka and colleagues (2009) tried to guide a sample of Japanese white-collar men to a healthier lifestyle. The study protocol, lasting 24 weeks with two balneotherapy sessions per week and twelve lifestyle interventions, encompassed physical exercise, dietary and psychological daily-living counselling for the control group (*n* = 21) and physical exercise, dietary and psychological practical interventions for the intervention group (*n* = 22). Sleep was assessed by asking the number of slept hours per night. At the end of the intervention, the study protocol failed to highlight any significant increase in the amount of hours slept (Kamioka et al., 2009).

The use of balneotherapy could also be called into question to solve or give relief in stressful situations. Indeed, the last study of this section engaged 108 German gardeners, farmers, and foresters for intervention in stress management. The protocol incorporated sessions of stress management intervention, relaxation techniques, physical exercise and balneotherapy (thermal water immersion), lasting 12 days with alternation of the different intervention typologies. Particularly for balneotherapy, the total number of sessions was four, and each balneotherapy session lasted 20 min. The authors focused on insomnia through the Insomnia Severity Index rather than sleep quality. Insomnia improved at the follow-up (ninth month), and the proportion of subjects with severe or moderate insomnia decreased (Stier-Jarmer et al., 2020).

#### 4.2.2 Balneotherapy and in-pool physical exercise

The three studies encompassing this section (Table 4) were set with patients affected by fibromyalgia (Altan et al., 2004; Maindet et al., 2021) and ankylosing spondylitis (Bestaş et al., 2022). The first two set the physical exercise intervention in thermal water pools (Altan et al., 2004; Maindet et al., 2021), while the latter proposed the physical exercise intervention in a swimming pool (Bestaş et al., 2022).

**TABLE 4:** Balneotherapy and physical exercise–Balneotherapy and in-pool physical exercise.

Study	N; population type	Age	Study design	Intervention	Comparison(s)	Sleep assessment	Follow-up(s)	Sleep results	Significant changes
Altan et al. (2004)	50; women with fibromyalgia	43.1 (IG) 43.9 (CG)	RCT	PBPT (*n* = 25) 36 sessions; 1 treatments per day; 35 min	BT (*n* = 25) 36 sessions; 1 treatment per day; 20 min	Hamilton Depression Scale; primary outcome	Study end; 6th month	The IG improved the sleep quality significantly after both the follow-ups; the CG significantly improved the sleep quality only after the first follow-up	Yes
Maindet et al. (2021)	218; patients with fibromyalgia	49.8	RCT	SPA therapies (*n* = 108) 18 sessions; 72 treatments per day; 2 h	Regular treatment (*n* = 110)	PSQI; secondary outcome	1st month; 3rd month; 6th month; 9th month; 12th month	No changes in sleep quality	No
Bestaş et al. (2022)	60; patients with ankylosing spondylitis	42.3 (BT) 38.6 (PE)	RCT	BT (*n* = 30) 20 sessions; 20 min	PE (*n* = 30) 20 sessions; 60 min	PSQI	Study end; 3rd month	Sleep improved in both groups in the total score and most but not all domains	Yes

IG, intervention group; CG, control group; RCT, randomised clinical trial; BT, balneotherapy; PBPT, pool-based physical therapy; PSQI, pittsburgh sleep quality index; PE, physical exercise.

In the first study, the participants were stratified into two groups: the intervention group (thermal-pool and on-land physical exercise; *n* = 24) and the control group (balneotherapy with water immersion; *n* = 22). Both interventions lasted 35 min, and the protocol consisted of thirty-six sessions. Sleep was assessed through some questions of the Hamilton Depression Scale, which improved in the intervention group from baseline to both twelfth- and twenty-fourth-week follow-up, whereas it improved only at the twelfth-week follow-up in the control group. However, the comparison between the two groups did not show any significant differences, and the authors were not able to affirm the superiority of physical exercise over balneotherapy and vice-versa.

Opposite results are reported by Maindet and colleagues (2021). The recent, larger, and longer-lasting study involved 220 women with fibromyalgia in eighteen balneotherapy sessions (hydromassage baths, hydro-mineral mud applications, body jet showers, water affusion massages, collective exercise in a mineral water pool) lasting 2 h. Unlike the previous study and notwithstanding the improvements in pain and anxiety, sleep quality, assessed with PSQI, was superimposable between the baseline and the follow-up time points (after 3, 6, and 9 months). Thus, sleep did not change after the balneotherapy protocol (Maindet et al., 2021). As happened in other studies, the authors did not comment on sleep results. However, it could be speculated that the discrepancies with the previous results by Altan and colleagues 2004 could be traced to the characteristics of the tools used to assess sleep. Indeed, PSQI is more accurate in describing sleep by investigating seven sleep domains.

Finally, ankylosing spondylitis patients seemed to benefit equally from balneotherapy (20 sessions lasting 20 min each) and in-pool physical exercise (20 sessions lasting 60 min each). The PSQI final score, sleep latency, habitual efficiency, disturbances, use of sleep medications and daytime dysfunctions, but not sleep duration, improved either at the end of the protocol or at the 12th week of follow-up (Bestaş et al., 2022). The authors advanced several hypotheses aiming at explaining the beneficial effects of both balneotherapy and physical exercise. Physical exercise is assigned of analgesic and muscle relaxation effects (Anderson and Shivakumar, 2013), which are also shared by balneotherapy (Fioravanti et al., 2010), the benefits of which are reinforced by the mechanical, chemical, immunomodulatory and buoyancy thermal water’s effects (Nasermoaddeli and Kagamimori, 2005).

### 4.3 Possible mechanisms linking spa therapies and physical exercise to sleep

#### 4.3.1 Possible mechanism of spa therapies’ action on sleep

As previously mentioned, the positive consequences of balneotherapy on sleep are probably the results of the combination of thermal, mechanical and chemical factors (Nasermoaddeli and Kagamimori, 2005).

The application of hot water causes an increase in the skin body temperature, leading to generalized peripheral vasodilatation, which induces a reduction in systemic blood pressure and core body temperature (Fioravanti et al., 2011). Moreover, vasodilatation improves the removal of cytokines and toxins involved in the inflammatory process of pathological conditions. In fact, it has been suggested that balneotherapy has an inhibitory effect on the production and release of interleukin-1 (IL-1), prostaglandin E_2_ (PGE_2_), and leukotriene B_4_ (LTB_4_), essential mediators of inflammation and pain (Ardıç et al., 2007).

Hot stimuli, which also produce analgesia on nerve endings and increase the pain threshold, cause a reduction of pain and muscle relaxation (Melzack and Wall, 1965; Fioravanti et al., 2011). All these mechanisms could lead to favourable conditions for sleep induction and improvement.

Thermal stress induces the same reactions also in the endocrine system, particularly with the release of adrenocorticotropic hormone (ACTH), cortisol, and growth hormone (GH) (Kuczera and Kokot, 1996). Concerning the moment the treatment is carried out, these therapies could lead to a disruption of hormone circadian rhythm, especially if done in the evening hours, resulting in sleep disturbance. This is why the balneotherapy, particularly muds, should be mainly carried out in the first part of the day. In this way, the analgesic effect of these hormones can be enhanced. Furthermore, the heat treatment leads to an increase in plasma levels of beta-endorphins, producing an analgesic and antispastic effect with a consequently positive effect on nocturnal sleep (Cozzi et al., 1995).

The mechanical effects of immersion allow the subjects to mobilize joints and strengthen them with less effort (O’hare et al., 1985). Improving the musculoskeletal conditions and, therefore, the pain could influence sleep characteristics.

Regarding the chemical effects, they act mainly on the skin by producing physiological responses such as vasodilatation in the microcirculation, an analgesic influence on pain receptors and inhibition of the immune system (Matz et al., 2003; Nasermoaddeli and Kagamimori, 2005), all conditions favouring sleep.

Moreover, an atmosphere of relaxation such as that of the thermal environment should result in less psychological tension and a night of more restful sleep (Sukenik et al., 1999; Bender et al., 2005; Fioravanti et al., 2011).

#### 4.3.2 Possible mechanism of physical exercise’s action on sleep

Physical exercise could promote and influence sleep parameters through either physical actions or hormone impacts (Roveda et al., 2011; Vitale et al., 2017).

Physical exercise can mimic the body cooling in preparation for sleep. Indeed, body temperature increases during exercise, and afterwards, it drops through dissipation mechanisms, including peripheral vasodilation. The similarities between the changes in body core temperature during/after physical exercise and before falling asleep could help signal to the brain that it is time to fall asleep (Driver and Taylor, 2000). Furthermore, sleep is an instrument to preserve energy use and promote energy conservation. Since after physical exercise, a larger energy amount is depleted, more sleep or a night of more restorative sleep is needed to restore energy balance and preserve the energy extent (Driver and Taylor, 2000).

Stress and depression can intrude on the ability to fall asleep and maintain sleep; endorphins are hormones able to improve mood and sleep quality (National Sleep Foundation, 2019), and one of the most known is the Brain-Derived Neurotrophic Factor (BDNF). Regular physical exercise is well known to raise endorphin secretion and BDNF concentration during exercise bouts (Uchida et al., 2012; Du et al., 2015). Indeed, in depressed subjects practising physical exercise regularly, BDNF concentrations are regularized, and depression symptoms attenuated (Monteiro et al., 2017). From a different point of view, the relation physical exercise-BDNF-Sleep can also be described as Sleep-BDNF-physical exercise. In other words, the loss of sleep quality leads to higher stress levels, stimulating cortisol secretion and, simultaneously, suppressing BDNF production. Diminished BDNF production exposes to a great depression vulnerability; however, physical exercise is considered one of the tools able to improve BDNF balance, stress, and sleep (Monteiro et al., 2017).

Physical exercise is also involved in decreasing body mass and fat mass. Overweight or obese subjects could sometimes be described as bad sleepers or suffering from several sleep problems, including obstructive sleep apnoea. In this view, Farnsworth and colleagues’ (2015) and Nam and colleagues (2016) showed an improvement in sleep quality by reducing body weight through physical exercise (Farnsworth et al., 2015; Nam et al., 2016). Some biological explanations could be found in the abnormal responses in ghrelin, leptin, and orexin after sleep restriction. On the one hand, obesity risk increases with a chronic sleep duration of <6 h. On the other hand, the lack of equilibrium between these metabolic hormones is responsible for overeating and increasing body mass. Physical exercise, influencing sleep quality and duration, could also be responsible for the re-equilibrium of these hormones (Atkinson and Davenne, 2007; Leproult and Van Cauter, 2009).

Some cytokines regulating the inflammatory process are also intricated in the sleep-wake cycle regulation, and, principally, the IL-6 and the Tumour Necrosis Factor *α* (TNF-α) are sensitive to sleep homeostasis (Shearer et al., 2001). Sleep loss or total sleep restriction and the presence of sleep problems are two factors elevating pro-inflammatory cytokines (IL-1β, IL-6, and TNF-α) and marker (CRP) expression during the night (Lekander et al., 2013; Zielinski et al., 2014; Irwin et al., 2016). In such a situation of chronic sleep restriction, physical exercise could act directly and indirectly to reduce the pro-inflammatory circulating factors. Firstly, physical exercise can directly decrease IL-6, TNF-α, and other circulating pro-inflammatory cytokines (Suzuki, 2019); secondly, physical exercise can reduce them by improving sleep duration (Chennaoui et al., 2020).

## 5 Overall and final considerations

After analyzing the included articles, balneotherapy associated with other spa treatments and physical exercise seems to be effective in improving the self-perceived sleep quality. Indeed, fifteen out of twenty studies (75%) described improvements in self-perceived sleep quality, which could even last several months from the end of the study. Excluding the four articles with VAS sleep assessment, twelve out of sixteen studies (75%) reported significant sleep changes. The remaining studies show neither sleep quality improvements nor worsening; thus, balneotherapy and physical exercise could be attributed, at least, to a protective effect in avoiding sleep deterioration.

Study protocols differ significantly from one to another. One of the most changing aspects of the study protocol is the duration of the whole intervention. It appears that 4 weeks of treatments are necessary to elicit significant and long-lasting sleep improvements when these are assessed with an appropriate sleep evaluation tool.

The intervention protocol gathering balneotherapy and exercise showed more lasting and incisive effects than other treatments or balneotherapy and exercise alone. Even though a meta-analysis was not performed, some considerations about the benefits of merging balneotherapy and physical activity could be advanced. Indeed, there are eight studies proposing balneotherapy and physical exercise, while those offering balneotherapy alone are five. Thus, it could be supposed that the combined effects of balneotherapy and physical exercise are preferred and perceived as better than balneotherapy alone. Furthermore, three out of five studies (60%; one used VAS) reported sleep improvements after balneotherapy alone, whereas seven out of eight studies (87.5%; one used VAS) improved sleep by combining balneotherapy and physical exercise. Additionally, the intervention groups (usually balneotherapy + physical exercise interventions) showed more significant sleep improvements than the control or comparison groups. It could be speculated that balneotherapy and exercise could sum up their effects in ameliorating some processes in influencing sleep, such as inflammation, pain relief, body temperature regulation, stress and anxiety, cortisol secretion and relaxation.

Regarding the combination of balneotherapy with other spa treatments or physical exercise, some hypotheses could be drawn on the effectiveness of the two therapeutic solutions. Also in this case, the solution balneotherapy + physical exercise collected more studies (ten) than the solution balneotherapy + spa treatments (six). In particular, the first solution mostly involved patients with physical and bodily impairments or pathologies, whereas the second was mainly administered to healthy subjects and patients with mental disorders. This could lead to the supposition that the combination between balneotherapy and other treatments could depend on the pathological situation. On the one side, patients suffering from pain and movement impairments could benefit more from the balneotherapy + physical exercise solution; on the other side, generally healthy subjects and patients with mental disorders could get more relief and relaxation from the combination of balneotherapy + other spa treatments.

However, apart from the five studies included in the section *Balneotherapy–thermal water immersion alone*, in which balneotherapy was the only administrated treatment, it is difficult, if not impossible, to discern the effects of balneotherapy on sleep isolated from the other treatments. Balneotherapy, spa and physical treatments, and exercise appear as a miscellany that positively influences self-perceived sleep quality. The peaceful and pleasant context of thermal centres could have probably favoured relaxation and the consequent sleep improvements.

The difficulties in discerning the effects of balneotherapy from physical exercise on sleep could also depend on the variety of the population types, which reflect different sleep problems and solutions. Since sleep is a secondary outcome in most studies, a detailed description of sleep problems is missing. However, notwithstanding the differences in the aetiology of the pathologies, some of them show similar characteristics, which allow tracing comparable traits in sleep problems and their way towards relief. Considering the included musculoskeletal pathologies (fibromyalgia, osteoarthritis, ankylosing spondylitis, etc.), it is well known and reported that in this class of pathologies, pain could affect sleep, and, as recently reported, deteriorated sleep could increase pain sensitivity (Husak and Bair, 2020). The reduction in pain sensitivity thanks to balneotherapy, spa therapy, and exercise may have also promoted more restful sleep. Thus, it could be speculated, as it is sometimes advanced in some included articles, that pain relief could lead to better sleep, which, in turn, could decrease pain sensitivity thanks to the bidirectional link between pain and sleep (Husak and Bair, 2020). Also in depression, mental and cognitive disorders, and burnout syndrome, a bidirectional nature between sleep and the pathology is highlighted. On the one side, sleep deprivation, disturbances, or insomnia could alter the normal rest-activity circadian rhythm and other circadian rhythms (Montaruli et al., 2021), the chronic altered conditions of which could lead to several mental disorders. On the other side, mental disorders, flanked or not by pharmacological treatments, could alter the normal sleep-wake cycle (Fang et al., 2019). The spa’s relaxing atmosphere and various treatments that engaged patients with mental illnesses may have favoured relaxation and a re-synchronization of the circadian rhythm, promoting better sleep. Data on these suppositions are not available in the studies; however, the alteration of the circadian rhythms and the importance of its re-establishment to recover from mental pathologies and sleep disorders are well known in literature (Pandi-Perumal et al., 2020; Riemann et al., 2020). Relaxation could also be called into question in the studies involving healthy participants, whose sleep improvements are not traceable in the amelioration of specific aspects of the pathology as seen for the two previous pathology classes. Indeed, relaxation strategies have already been adopted to improve sleep quality (Morin et al., 2006; Jane et al., 2011; Blanaru et al., 2012; Tan et al., 2021).

None of the studies investigated, demonstrated, or exactly described physiological processes, linkages, or cause-effects relationship between balneotherapy and sleep changes during and after treatments. Furthermore, none of the investigated variables (e.g., core body temperature, pain, stress level, cortisol, inflammation) has been correlated or investigated in their relationship with sleep. Considering the importance of restful and fulfilling sleep (Grandner, 2017), future studies will have to shed light on the possible mechanisms linking physiological changes during balneotherapy with sleep.

In this context, since sleep is assigned to an intrinsic circadian rhythm, considering the timing and the time of day of balneotherapy, spa treatments and exercise practice is essential to elucidate their effects on sleep. For example, the potentially predisposing lowering of body temperature (resulting from hot thermal water immersion) for sleep start could be solely accomplished if baths are taken in the late afternoon and not during the morning. Focusing on the multiple ways physical exercise could influence sleep and align with the above, physical exercise practice should also be preferred during the afternoon (Driver and Taylor, 2000). However, some cause-effect linkages between exercise and sleep (e.g., energy expenditure and body restoration) may be reached even when exercise is practised early in the day (Driver and Taylor, 2000; Uchida et al., 2012). Conversely, mud packs could find their better application timing in the morning since some studies reported increased cortisol levels after mud applications (Tanizaki et al., 1993; Ortega et al., 2017). The rise in cortisol levels could favour awakening and have adverse effects on promoting and maintaining sleep (Pruessner et al., 1997; Nader et al., 2010). The included studies failed to report the exact time for baths, mud pack application, physical exercise sessions, and treatments. This situation prevents us from finding an explanation of the relationship between treatment times and changes in sleep quality.

The results of the current review should be seen in light of its strengths and limitations. The former includes the topic’s novelty since it is the first that evaluates sleep quality in the balneotherapy context. Among the latter should be listed the heterogeneity of the study protocols, spa and balneotherapy treatments, sleep assessment methods (results obtained with the VAS method should be taken with a grain of salt), and the inclusion of different population types, including both healthy and unhealthy participants, who were affected by different pathologies. The miscellany of pathologies makes the analysis of the relief of sleep disturbances more torturous; however, some general considerations have been hypothesized. The dissimilarities in the sleep assessment methods lead to heterogeneity in the evaluated sleep dimensions or parameters. This circumstance prevented us from finding a common dimension of sleep for all the articles, which could have been assumed as a comparator parameter to evaluate balneotherapy and physical exercise effects. Finally, publication bias could not be excluded; however, the nature of the systematic review and the inclusion of non-randomized clinical trials prevented the calculation of publication bias.

## 6 Conclusion

The results of the current review demonstrate that balneotherapy in concomitance or not with exercise shows an effect on sleep, particularly in improving self-perceived sleep quality. However, due to the heterogeneity in the study protocols and administrated treatments, it has been impossible to markedly discern the effect of a single treatment and affirm the superiority of one intervention protocol. The relaxing atmosphere and the action of thermal water with its mechanical and chemical effects in relieving pain, decreasing inflammation, and influencing cortisol secretion could all be eligible pathways influencing sleep. However, future balneotherapy studies should focus on better explaining the likely-candidate physiological mechanisms affecting sleep quality. Furthermore, the lack of objective sleep assessments would suggest their involvement in the future.

## Data Availability

The original contributions presented in the study are included in the article/Supplementary Material, further inquiries can be directed to the corresponding author.
