# Supplementary material for: Sleep and spa therapies: What is the role of balneotherapy associated with exercise? A systematic review
Source: Front Physiol. 2022 Aug 10;13:964232. doi: 10.3389/fphys.2022.964232 (PMC9399348; doi:10.3389/fphys.2022.964232)
Supplement: Supplementary file 1 [file Image1.pdf]

Supplementary Material

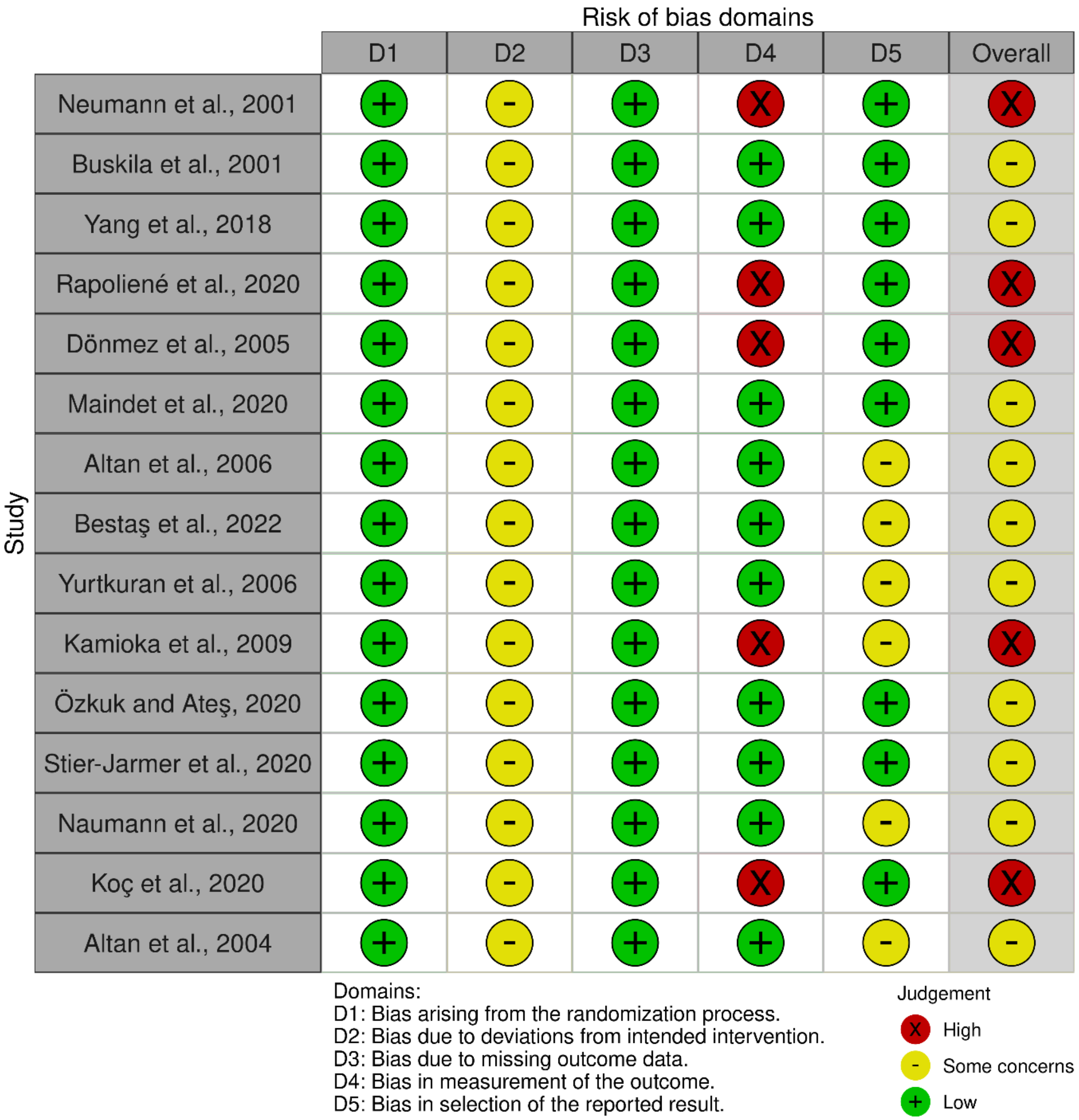

Supplementary Figure 1. Representation of the bias evaluation and results of the randomised clinical trials, analysed with the RoB-2 toll (Sterne et al., 2019) and figured out with the *robvis* tool (McGuinness and Higgins, 2021)

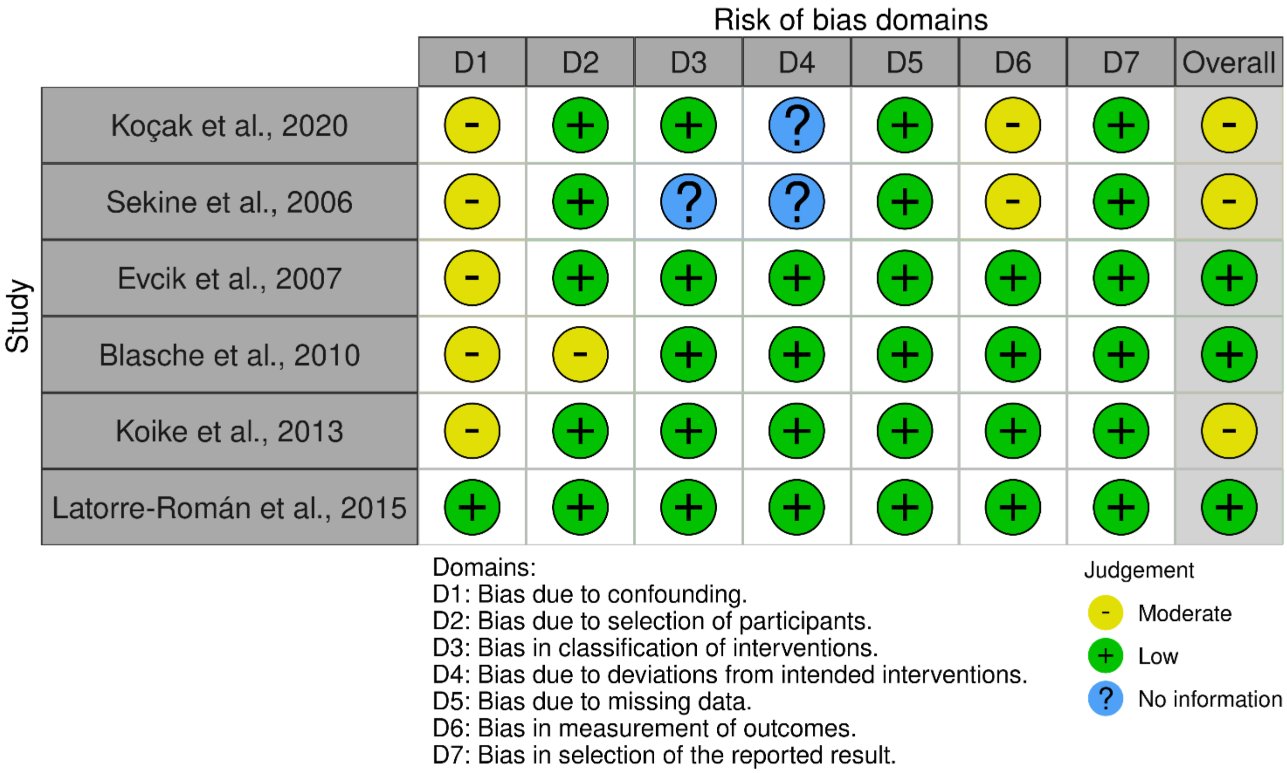

Supplementary Figure 2. Representation of the bias evaluation and results of the non-randomised intervention studies, analysed with the ROBINS-I toll (Sterne et al., 2016) and figured out with the *robvis* tool (McGuinness and Higgins, 2021)
